# Anti-Inflammatory Potential of Phenolic Compounds Isolated From *Entada africana* Guill. & Perr. Used in the Republic of Benin

**DOI:** 10.3389/fphar.2022.931240

**Published:** 2022-06-30

**Authors:** Nonvignon Murielle Codo Toafode, Peter Marquardt, Virgile Ahyi, Karin Fester, Verena Spiegler, Cica Vissiennon

**Affiliations:** ^1^ Inter-Regional University of Industrial Engineering Biotechnologies and Applied Sciences, IRGIB Africa University, Cotonou, Benin; ^2^ Institute of Medical Physics and Biophysics, Leipzig University, Leipzig, Germany; ^3^ Faculty of Natural and Environmental Sciences, Zittau/Görlitz University of Applied Sciences, Zittau, Germany; ^4^ Institute for Pharmaceutical Biology and Phytochemistry, University of Münster, Münster, Germany; ^5^ Repha GmbH Biologische Arzneimittel, Langenhagen, Germany

**Keywords:** *Entada africana*, anti-inflammatory effect, Republic of Benin, traditional medicine, phenolic compounds

## Abstract

In West African medicine, *Entada africana* Guill. & Perr. from the family of Fabaceae is used to treat inflammatory conditions in the management of fractures, wounds, and sprains in the northern region of the Republic of Benin. The aim of the present study was to isolate and elucidate phenolic compounds from a hydroalcoholic leaf extract from *E. africana* and to identify compounds with anti-inflammatory activity *in vitro*. Eleven compounds were purified from three fractions, which have shown strong to medium anti-inflammatory activity. The isolated compounds were characterized by HRESI-MS and NMR methods as gallic acid (**1**), ethyl gallate (**2**), 5,7-dihydroxychromen-4-one (**3**), 3′,4′,7-trihydroxyflavone (**4**), dihydrokaempferol-7-*O*-glucoside (**5**), catechin (**6**), quercetin-3-*O*-[β-apiosyl-(1‴→2″)-β-glucoside] (**7**), quercetin-3-*O*-glucoside (**8**), naringenin-7-*O*-glucoside (**9**), aromadendrin (**10**), and myricetin-3-*O*-glucoside (**11**). Nine of the major phenolic compounds were tested using TNF-α stimulated human keratinocytes (HaCaT) as skin inflammation model to identify molecules, which may explain the use of the plant leaves as an anti-inflammatory remedy by assessing the release of proinflammatory cytokines IL-8 and IL-6. The hydroacoholic leaf extract of *E. africana* exerted a medium inhibitory effect on the release of IL-8. 3′,4′,7-trihydroxyflavone, aromadendrin, dihydrokaempferol-7-*O*-glucoside and ethyl gallate demonstrated a strong to medium effect on the release of IL-6. For the release of IL-8, 3′,4′,7-trihydroxyflavone demonstrated a medium activity. This study provides for the first time a detailed screening of phenolic compounds occurring in the hydroethanolic leaf extract of *E. africana*. Additionally, it is shown that *E. africana* contains active compounds which may justify its traditional medicinal use as an anti-inflammatory remedy to treat inflammatory and pain-related skin conditions in the Republic of Benin.

## 1 Introduction

In African medicine, plants have been frequently used for the management of inflammatory and pain-related conditions such as wounds, joint inflammation, inflammatory skin disorders, and other inflammatory diseases (Maione et al., 2016; Oguntibeju, 2018). While an acute inflammation constitutes a vital response to an injury in all tissues, leading to the recruitment of inflammatory cells to eliminate the injurious agent and initiating a repair phase, an excessive or prolonged inflammatory response results in increased tissue injury and poor healing (Eming et al., 2007; Koh and DiPietro, 2011). The use of medicinal plants for inflammatory and pain-related skin conditions based on ethnomedicinal evidence emphasizes the importance of studying their chemical content to identify those that could be responsible for an observed therapeutic effect. Among these plants, *Entada africana* is described in several African cultures for its numerous ethnomedicinal uses (Yusuf and Abdullahi, 2019). It is a small and edible tree from the family of the Fabaceae which grows in the savannah and has been identified in different sub-Saharan African countries (Orwa et al., 2009; Baidoo et al., 2019). The plant is known to possess antiplasmodial, antimicrobial, anti-rheumatism, hepatoprotective, antioxidant, anti-inflammatory, and wound healing properties (Owona et al., 2013; Baidoo et al., 2019). In Burkina Faso, it is used for the treatment of diabetes, diarrhea, and hypertension. Due to the intense exploitation of its roots in this country it has appeared as an endangered species (Haidara et al., 2016). In the northern region of the Republic of Benin, the plant is used for the treatment of pain-associated conditions in the traditional treatment of wounds, fractures, and sprains (Vissiennon et al., 2011; Toafode et al., 2019). Apart from its ethnomedicinal uses, different substances have been identified in different parts of the plant. The roots have been widely investigated for different classes of compounds. The roots appeared to contain triterpene saponins, polysaccharides and β-sitosterol (Diallo et al., 2001; Cioffi et al., 2006; Shinkafi et al., 2015; Hassan et al., 2018). Additionally, two flavonoids (apigenin and robonetin) were identified in a CHCl_3_/MeOH root extract (Germanò et al., 2015). Active chemicals were identified from the stem bark fractions as hepatoprotective in synergy with *Khaya grandifoliola* (Njayou et al., 2016). Betulin with a moderate antibacterial activity has also been extracted from the stem bark of *E. africana* (Kwaji et al., 2018). Concerning pharmacological properties, a CH_2_Cl_2_ extract from the stem bark exerted *in vitro* anti-inflammatory activity in a lipopolysaccharide-induced inflammation in macrophages (Owona et al., 2013). Macerates of leaves and stem bark demonstrated a great potential for wound healing in animal models (Baidoo et al., 2019). However, only a few compounds present in the plant’s leaves are known. Our study aimed at investigating the anti-inflammatory potential *in vitro* and characterizing the phenolic profile of a hydroalcoholic leaf extract of *E. africana* in order to identify active compounds. Thus, TNF-α stimulated human keratinocytes (HaCaT) were used as a skin inflammation model to identify molecules, which may explain the use of the plant’s leaves as an anti-inflammatory remedy.

## 2 Materials and Methods

### 2.1 General Chemicals

If not stated otherwise, all solvents for extraction and fractionation were purchased from VWR International GmbH, Darmstadt, Germany. All solvents were of analytical grade. DMSO was obtained from Sigma-Aldrich, Steinheim, Germany, 3-(4,5-dimethylthiazol-2-yl)-2,5-diphenyltetrazolium (MTT) from Sigma-Aldrich, Taufkirchen, Germany, sodium dodecyl sulfate (SDS) from ThermoFisher Scientific, Rockford, United States, and 2-aminoethyl diphenyl borate from Carl Roth, Germany.

### 2.2 Plant Material Collection

The leaves of *E. africana* were harvested in December 2015 in Bassila in the department of Donga (9°41′59.99″ N/1°39′59.99″ E as coordinates) in the Republic of Benin. Samples were authenticated and collected with the assistance of a botanist, and a voucher specimen was deposited at the National Herbarium of the University of Abomey-Calavi (No.: YH 455/HNB). Exportation allowance for pharmacological testing and chemical characterization was obtained from the Ministry of Agriculture, Livestock and Fisheries, Republic of Benin.

### 2.3 General Analytical Techniques

Thin layer chromatography was performed on TLC Silica Gel 60 F254 plates (Merck KGaA, Darmstadt, Germany). Samples were prepared in the range of 1–2 mg per mL, and 4 µL of each sample were applied as 10 mm bands by means of the CAMAG Automatic TLC Sampler 4 (AST4). The chromatograms were developed in a CAMAG chamber over 8 cm running distance with prior chamber saturation. The system water: formic acid: ethyl acetate (5:5:90, *v*/*v*/*v*) constituted the mobile phase. The developed plates were allowed to air dry, and chromatograms were detected at 254 nm using CAMAG TLC Visualizer 2 operated under the software vision CATS 2.5. In addition, derivatization was performed with natural product reagent (2-aminoethyl diphenyl borate) as a detecting reagent for flavonoids and chromatograms were evaluated at 366 nm.

Analytical UPLC for fractionation and purity control was performed using Acquity™ Ultra Performance LC, PDA λe Detector and QDa™ Detector, autosampler, in-line degasser and Waters Empower 3^®^ Software (Waters, Milford, MA, United States). Separation was performed on an RP-18 stationary phase (HSS T3, 1.8 µm, 2.1 × 100 mm) with the following binary gradient (A: water with 0.1% formic acid; B: acetonitrile with 0.1% formic acid) at a flow rate of 0.5 mL/min: *t*_0min_ 2% B, *t*_0.5min_ 2% B, *t*_5.5min_ 5% B, *t*_6.5min_ 12% B, *t*_30min_ 25% B, *t*_41.5min_ 70% B, *t*_46min_ 95% B to *t*_50min_ 100% B. Samples were generally prepared at a concentration of 1–2 mg/mL.

Chromatographic separations for LC-HRESI-MS were performed on a Dionex Ultimate 3000 RS Liquid Chromatography System on a Dionex Acclaim RSLC 120, C18 column (2.1 × 100 mm, 2.2 µm) with a binary gradient (A: water with 0.1% formic acid; B: acetonitrile with 0.1% formic acid) at 0.4 mL/min: *t*_0min_ 0% B, *t*_0.4min_ 5% B, *t*_9.9min_ 100% B, *t*_15min_ 100% B. The injection volume was 2 µL. Eluted compounds were detected using a Dionex Ultimate DAD-3000 RS over a wavelength range of 200–400 nm and a Bruker Daltonics micrOTOF-QII time-of-flight mass spectrometer equipped with an Apollo electrospray ionization source in positive mode at 4 Hz over a mass range of *m/z* 50–1,500 using the following instrument settings: nebulizer gas nitrogen, 4 bar; dry gas nitrogen, 9 L/min, 220°C; capillary voltage 4500 V; end plate offset -500 V; transfer time 100 µs; collision gas nitrogen; collision energy and collision RF settings were combined to each single spectrum of 1,250 summations as follows: 624 summations with 80 eV collision energy and 130 Vpp + 313 summations with 16 eV collision energy and 130 Vpp + 313 summations with 16 eV collision energy and 130 Vpp. Internal dataset calibration (HPC mode) was performed for each analysis using the mass spectrum of a 10 mM solution of sodium formiate in 50% isopropanol that was infused during LC reequilibration using a divert valve equipped with a 20 µL sample loop.

NMR spectra were recorded at 299 K on an Agilent DD2 spectrometer (Agilent Technologies, Santa Clara, United States ) at 600 MHz (^1^H) or 150 MHz (^13^C). Samples were dissolved in methanol-*d*4 (4.87; 49.00 ppm) and chemical shifts were referenced to the respective residual solvent signals.

### 2.4 Extract Preparation

The leaves of *E. africana* were oven dried at 40°C and ground to obtain a fine powder for further extraction. The powdered material (200 g) was defatted with petroleum ether solvent using a Soxhlet apparatus. The defatted plant material (183.3 g) was then extracted with ethanol:water (2 L, 1:1 *v*/*v*) three times, resp. by Ultra Turrax^®^ (IKA T25, Staufen, Germany) at 13,000 rpm for 10 min. The extract was centrifuged for 5 min at 3,000 × *g*, supernatants were collected and lyophilized after evaporation of the organic solvent. The dried extract [yield: 26.6 g, 13.3% (*w*/*w*) of the dried plant material] was stored at −20°C for further analysis.

### 2.5 Fractionation of *E. africana* Ethanolic Extract

#### 2.5.1 Column Chromatography on Sephadex^®^ LH-20

The hydroalcoholic extract of *E. africana* (20.02 g) was dissolved in ethanol-water (1:1 *v*/*v*) and loaded onto a chromatography column packed with Sephadex^®^ LH-20 as stationary phase. The fractionation of the extract was carried out using a step gradient from ethanol, methanol/water (1:1 *v*/*v*), methanol to acetone/water (7:3 *v*/*v*), and the fractions collected were monitored by TLC. Fractions were combined according to their fingerprints to obtain 25 fractions that were analyzed by UPLC-PDA.

#### 2.5.2 Medium Pressure Liquid Chromatography of FC4

MPLC technique used for preparative separation of organic compounds was applied for subfractionation and isolation of compounds from FC4. 1 g of the fraction was diluted in 8 mL methanol 30% (*v*/*v*) and loaded at the top of the column (36 × 500 mm) filled with RP18 material (18–32 µm, 100 Å; BESTA Technik, Wilhelmsfeld, Germany). A step gradient of methanol 10, 20, 50, 70, and 100% (*v*/*v*) was used at a flow rate of 4 mL/min. The fractions collected were analyzed by TLC and combined to yield 6 sub-fractions (FC4-I to FC4-VI). FC4-I was entirely composed of gallic acid (**1**, 9.9 mg: purity 100%).

#### 2.5.3 Isolation of Compounds

For the isolation of pure compounds from FC3, FC8 and sub-fractions FC4-II and FC4-III, the fractions were dissolved to 10 mg/mL in a mixture of acetonitrile and water resp. and 1,500 µL were injected for each run. Preparative HPLC was carried out using Quaternary Gradient Module 2545, Photodiode Array Detect or 2998, Autosampler 2707, Waters Prep Degasser and Waters Fraction Collector III. Software: Waters ChromScope v1.40 Beta (Waters, Milford, MA, United States). Stationary phase: Nucleodur^®^ C18 HTec, 5 µm, 250 × 21 mm, mobile phase: binary gradient of water (A) and acetonitrile (B). Ethyl gallate (**2**, 6.1 mg; purity 98%), 5,7-dihydroxychromone (**3**, 1.8 mg; purity 89%) and 3′,4′,7-trihydroxyflavone (**4**, 4.5 mg; purity 98%) were isolated from fraction FC3. Subfraction FC4-II yielded dihydrokaempferol-7-*O*-β-glucopyranoside (**5**, 4.4 mg; purity 91%) and catechin (**6**, 0.6 mg; purity 91%). Quercetin-3-*O*-[β-apiosyl-(1‴→2″)-β-glucopyranoside] (**7**, 3.4 mg; purity 99%), isoquercitrin (**8**, syn.: quercetin-3-*O*-β-glucopyranoside; 4.8 mg; purity 99%), prunin (**9**, syn.: naringenin-7-*O*-β-glucopyranoside; 63 mg; purity 100%) and aromadendrin (**10**, syn.: dihydrokaempferol; 6.3 mg; purity 98%) were obtained from fraction FC4-III. Finally, FC8 yielded myricetin-3-*O*-β-glucopyranoside (**11**, 0.6 mg: purity 87%), with further amounts of **8** (0.9 mg) and **9** (3 mg).

### 2.6 *In Vitro* Anti-inflammatory Activity Testing

#### 2.6.1 Cell Culture and Conditions

Immortalized human keratinocyte cells (HaCaT) (Boukamp et al., 1988; López-García et al., 2014) were purchased from CLS Cell Lines Service GmbH, Eppelheim, Germany and cultured in Dulbecco’s Modified Eagles Medium (DMEM, Biowest, Nuaillé, France) supplemented with 1% penicillin/streptomycin (P/S, Biowest), 10% fetal calf serum (FCS, Biowest) in cell culture flasks (Sarstedt Nümbrecht, Germany) at 37°C in a humidified atmosphere at 5% CO_2_. Experiments were conducted at a cell confluency of 80–90%. Cells were seeded at 5.0 × 10^4^/500 µL after detachment using trypsin-EDTA (0.05%) solution (Biowest), in 24 well plates and incubated for 24 h.

#### 2.6.2 *In Vitro* Inflammation Assay and Cytokine Quantification

The experiments were carried out according to Marquardt et al. (2020a), with some modifications to the protocol. After cell seeding and incubation, recombinant human TNF-α (40 ng/mL, Humanzyme, Chicago, IL, United States) was added to the cells and coincubated with plant extract, fractions (1–100 µg/mL), or pure compounds (0.1–100 µM) for 24 h. DMSO (0.0025%) as well as culture medium were applied as vehicle and untreated control respectively. Afterwards, supernatants were collected to assess the release of cytokines IL-8 and IL-6 as biomarkers of the anti-inflammatory response using ELISA (BD Biosciences, San Diego, CA, United States) according to the manufacturer’s instructions.

#### 2.6.3 Cell Viability Assay

Cell viability of the TNF-α stimulated HaCaT cells was tested with a subsequent MTT assay. Thus, after collecting the supernatant, cells were treated with an MTT solution (0.3 mg/mL in DPBS) and incubated for 2 h. The resulting formazan salt was dissolved with 500 µL SDS lysis buffer (20 g SDS, 40 mL DMF, ad 100 mL aqua dest. pH = 4,7). The control well is treated with the culture medium, and in another well, cells are lysed with triton. After an incubation overnight, the amount of formazan was quantified spectrophotometrically at 570 nm using an Infinite^®^ M 200 plate reader (TecanGroup Ltd. Mannedorf, Switzerland).

### 2.7 Statistical Analysis

Spectrophotometric data from cytokine release experiments and cytotoxicity testing were processed with Microsoft Excel version 2016. Statistical analysis was performed with GraphPad Prism (version 9.3.1). Interleukin amount and metabolic activity were normalized to the TNF-α stimulated untreated control (set as 100%). One-way analysis of variance (ANOVA) was used to evaluate the differences between the control and treatment groups. Values ****p ≤ 0.0001, ***p ≤ 0.001 **p ≤ 0.01, and *p ≤ 0.05 compared to TNF-α control were considered statistically significant. Half-maximal inhibitory concentrations (IC_50_) were computed based on the non-linear regression of the concentration–response plots. Maximum inhibition (MI) was calculated from normalized values and represents inhibition at 100 µg/mL for plant extract and fractions or at 100 µM for pure compounds.

## 3 Results

### 3.1 Fractionation and Bioactivity of *E. africana* Extract

**FIGURE 1:**
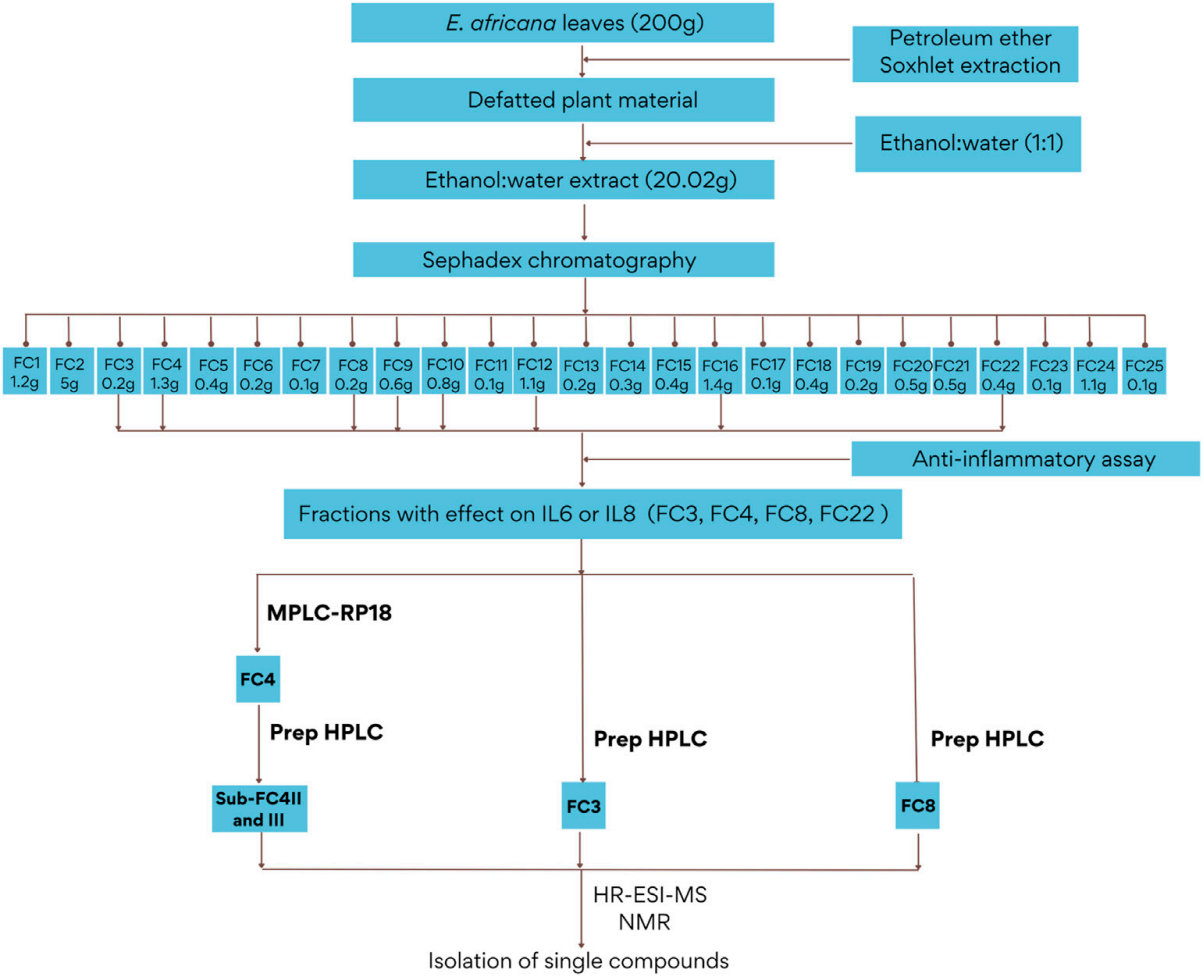
Isolation pattern for compounds 1–11 from *Entada africana* leaves extract.

The leaves of *E. africana* were defatted and extracted with ethanol-water (1:1 *v*/*v*) for further fractionation steps. Then, the extract was subjected to a fractionation which led to a total of 642 single fractions collected. Similar fractions were combined, resulting in 25 fractions (FC1—FC25) and screened for their phenolic profile by TLC and UPLC-PDA-MS. The fractionation pattern is presented in Figure 1 and TLC profiles of FC1 to FC25 can be found in the supplementary material (Figure 1). According to their phenolic profile, 8 fractions (FC3, FC4, FC8, FC9, FC10, FC12, FC16, and FC22) as well as the hydroalcoholic extract of *E. africana* (Ea) were tested for their anti-inflammatory properties *in vitro* using an immortalized keratinocyte cell line (HaCaT). The stimulation of HaCaT cells with TNF-α led to the secretion of interleukin IL-6 (3.4-fold vs untreated control, *p* < 0.05) and IL-8 (2.5-fold vs untreated control, *p* < 0.05). In addition, budesonide 10 mM used as a positive control reduced IL-6 and IL-8 release to 57.5 ± 4.7% and 44.4 ± 5.5% respectively (*p* < 0.05). The hydroalcoholic extract of *E. africana* inhibited the release of IL-8 to 48.1 ± 3.5% with an IC_50_ = 59.2 µg/mL, 95% CI (36.1–97.0) while there was no effect on the release of IL-6. Fractions FC3, FC4 and FC22 demonstrated a strong to medium activity on IL-6 release while IL-8 is strongly and moderately reduced respectively by FC22 and FC8. The remaining fractions (FC9, FC10, FC12, and FC16) did not affect the release of IL-6 and IL-8. All results are summarized in Table 1. Cell viability was reduced to 58 ± 1.9%, *p* < 0.0001 by the hydroalcoholic leaves extract compared to the negative control, while none of the fractions showed any reduction in cell viability up to 100 µg/mL.

**TABLE 1:** Summary of inhibitory effects of *Entada africana* leaf extract (Ea) and its fractions FC3, FC4, FC8, FC9, FC10, FC12, FC16, FC22 (1–100 µg/mL) on cytokine (IL-8, IL-6) release from TNFα-stimulated HaCaT cells.

Mediator	Interleukin-8 (IL-8)		Interleukin-6 (IL-6)	
Treatment	MI (%)	IC50 [95%CI] (µM)	MI (%)	IC50 [95% CI] (µM)
Ea	48.1 ± 3.5	59.2 [36.1–97.0]	—	—
FC3	29.9 ± 3.6	55.3 [36.9–82.8]	**70.4 ± 2**	**37 [20.8–65.7]**
FC4	—	—	56.2 ± 2.3	64.8 [18.9–222.9]
FC8	51.4 ± 1.9	36.9 [10.4–130.9]	35 ± 6.5	148.6 [0.01 - 2e6]
FC9	—	—	—	—
FC10	—	—	—	—
FC12	—	—	—	—
FC16	—	—	—	—
FC22	**71.7 ± 5.5**	**63.5 [10.1–401.7]**	57.8 ± 8.7	36.5 [7.1–187.2]

MI, maximum inhibition, IC_50_, half maximal inhibitory concentration.

The values in bold represent: Fractions with a strong inhibitory effect (≥70%).

### 3.2 Phenolic Compounds Isolated From *E. africana*

**FIGURE 2:**
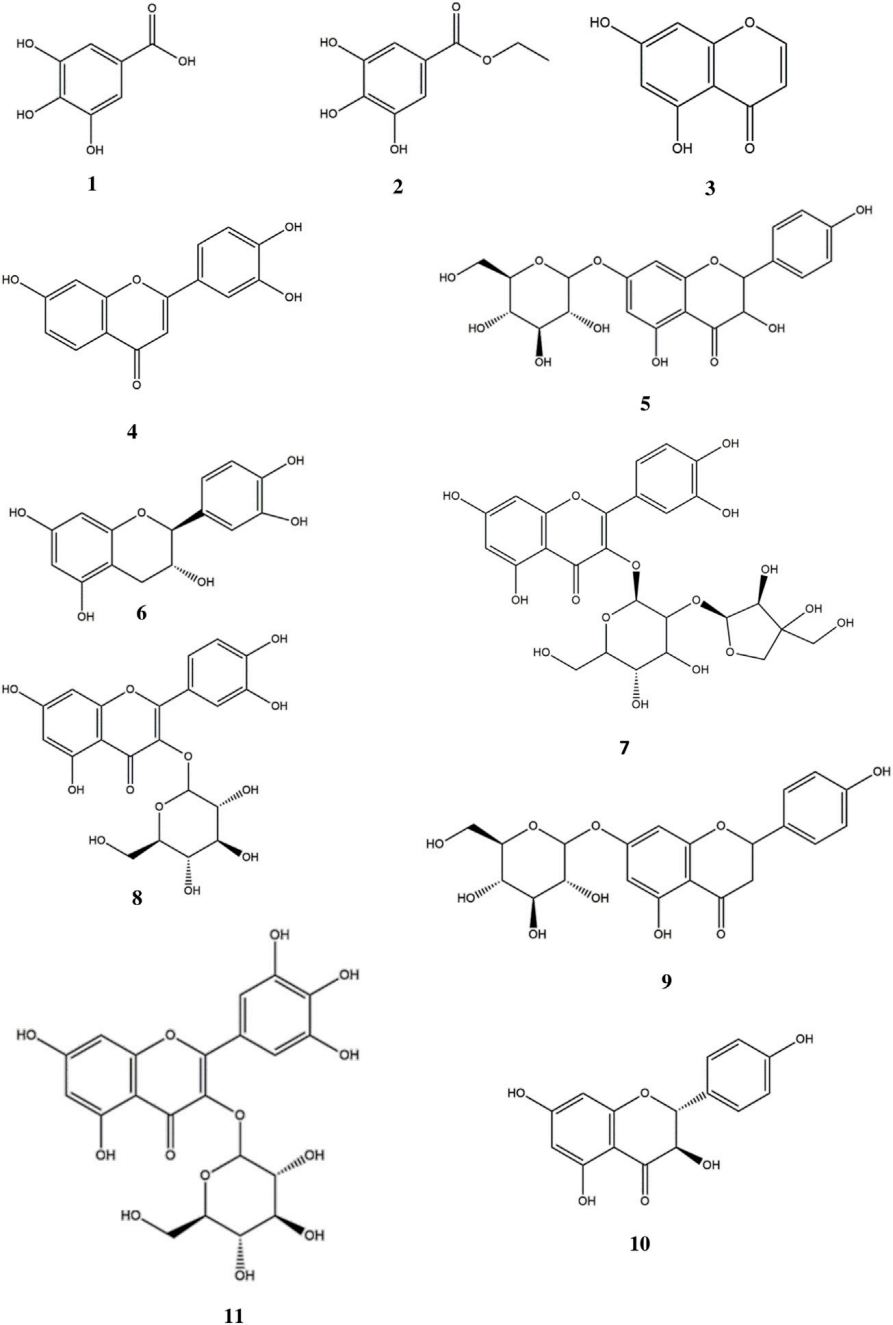
Phenolic compounds isolated from *Entada africana*: Gallic acid **(1)**, Ethyl gallate (**2**), 5,7-dihydroxychromone (**3**) and 3′,4′,7-trihydroxyflavone **(4)** from fraction FC3. Dihydrokaempferol-7-*O*- glucopyranoside (**5**) and catechin (**6**) from FC4-II, FC4-III yielded quercetin-3-*O*-[β-apiosyl-(1″→2″)-β-glucopyranoside] (**7**), isoquercitrin (**8**), prunin (**9**) and aromadendrin [syn. dihydrokaempferol (**10**)]. Myricetin-3-*O*-glucopyranoside (**11**) obtained from FC8.

Fractions FC3, FC4 and FC8 were selected for further fractionation due to their bioactivity in order to isolate phenolic compounds. Although fraction 22 has shown a significant inhibitory effect on the release of IL-6 and IL-8, its TLC and HPLC analysis revealed the presence of high molecular weight tannins. As these compounds can currently not be separated by common chromatographic methods, FC22 was not further fractionated. For fraction FC4, medium pressure chromatography on RP-18 stationary phase was performed leading to six subfractions (FC4-I to FC4-VI). Subfraction FC4-I was revealed to be a pure compound showing an *m/z* value of 171.0298 ([M + H] ^+^) which corresponds to gallic acid (**1**). The structure was further confirmed by NMR in comparison to published data (Wang et al., 2015). The isolation of compounds from fraction FC3 and FC8 as well as from subfractions FC4-II and FC4-III was carried out by preparative HPLC. Ethyl gallate (**2**) (Zhao et al., 2009), 5,7-dihydroxychromone (**3**) (Simon et al., 1994), and 3′,4′,7-trihydroxyflavone (**4**) (Júnior et al., 2008; Xu et al., 2018) were isolated from fraction FC3. Dihydrokaempferol-7-*O*-glucoside (**5**) (Li and Cui, 2014) and catechin (**6**) (Hori et al., 2018) were obtained from FC4-II. FC4-III yielded quercetin-3-*O*-[β-apiosyl-(1‴→2″)-β-glucopyranoside] (**7**) (Kasaj et al., 2001), isoquercitrin (**8**) (Kazuma et al., 2003), prunin (syn. naringenin-7-*O*-glucoside (**9**) (Turner et al., 2005), and aromadendrin (syn. dihydrokaempferol (**10**) (Lee et al., 2003; Han et al., 2007). Finally, myricetin-3-*O*-glucoside (**11**) (Kazuma et al., 2003) together with small amounts of **8** and **9** was isolated from FC8. Structures of the compounds are represented in Figure 2. All substances were identified by HR-ESI-MS and NMR in comparison to published data (see Supplementary Material).

### 3.3 Bioactivity of Phenolic Compounds Isolated From *E. africana*

**FIGURE 3:**
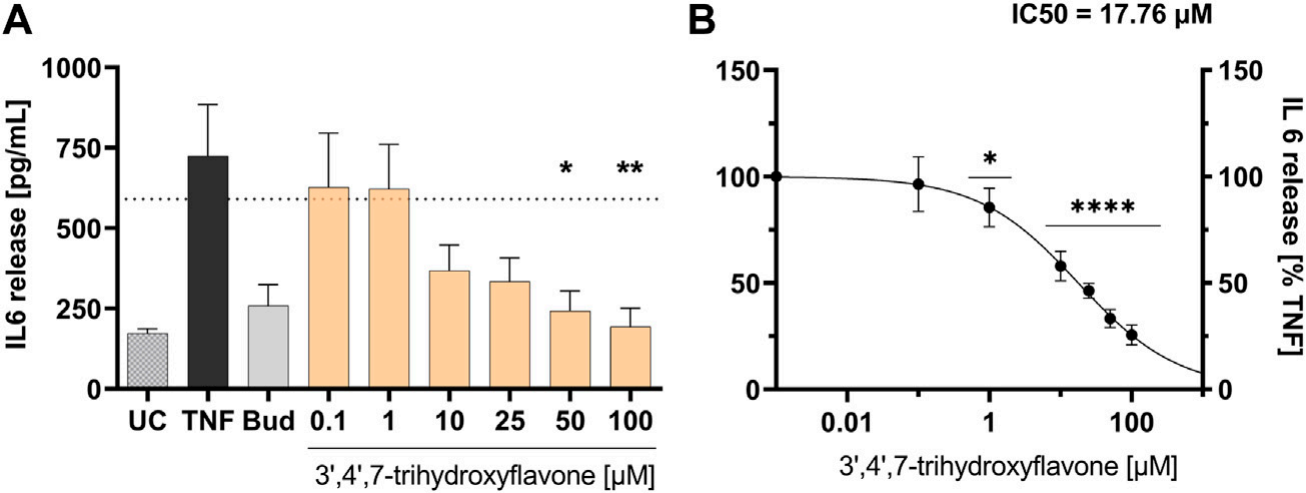
Influence of 3′,4′,7-trihydroxyflavone (1–100 µM) on TNF-α induced inflammatory mediator release from HaCaT cells **(A)**. Influence on IL-6 release [pg/mL]; **(B)** concentration response curve for the inhibition of IL-6 release [% TNF-α control]; UC = untreated control, TNF = TNF-α stimulation and untreated control, Bud = TNF-α stimulation and budesonide 10 mM. Mean ± standard error of the mean (SEM), *n* = 4–6, significant ****p ≤ 0.0001, **p ≤ 0.01, and *p ≤ 0.05 compared to TNF-α treated cells ( = 100%) in ordinary 1-way ANOVA.

Eleven phenolic compounds were identified and isolated from *E. africana* leaves hydroalcoholic extract. Compounds **2** to **5** and **7** to **11** were assessed for their *in vitro* anti-inflammatory effect. Since there is sufficient literature on compounds **1** and **6** as compounds with anti-inflammatory activity, no analysis was performed. The results reported in Table 2 indicate inhibitory effects of purified compounds isolated from FC3, FC4 and FC8. 3′,4′,7-trihydroxyflavone strongly reduced the secretion of IL-6 with a maximum inhibition of 74.4 ± 2%, IC_50_ = 17.8 µg/mL, 95% CI (4.5–69.6) (see Figure 3) followed by aromadendrin, dihydrokaempferol-7-*O*-beta-D-glucopyranoside and ethyl gallate which have a medium inhibitory effect. Additionally, 3′,4′,7-trihydroxyflavone moderately reduced the secretion of interleukin IL-8 to 40.2 ± 5.1% with IC_50_ = 126.2 µg/mL, 95% CI (0.04–4.1e5) (see detailed results in Table 2). Meanwhile, cell viability was reduced to 74 ± 1.8%, *p* < 0.001 at 100 µM for 3′,4′,7-trihydroxyflavone, while not affected by the remaining compounds tested.

**TABLE 2:** Summary of inhibitory effects of *Entada africana* compounds **2-5** and **7–11** (0.1/1–100 µM) from cytokine (IL-8, IL-6) release from TNFα-stimulated HaCaT cells.

Mediator	Interleukin-8 (IL-8)		Interleukin-6 (IL-6)	
Treatment	MI (%)	IC50 [95%CI] (µM)	MI (%)	IC50 [95% CI] (µM)
Ethyl gallate (**2**)	—	—	42.5 ± 4.6	10.9 [6.8–17.3]
5,7-dihydroxychromone (**3**)	—	—	—	—
3′,4′,7-trihydroxyflavone (**4**)	40.2 ± 5.1	126.2 [0.04–4.1e5]	**74.4 ± 2**	**17.8 [4.5–69.6]**
Dihydrokaempferol-7-*O*-beta-D-glucopyranoside (**5**)	—	—	59.3 ± 5.8	7.4 [2.6–20.9]
Quercetin-3-*O*-[β-apiosyl-(1‴-> 2″)-β-glucopyranoside (**7**)	—	—	—	—
Quercetin-3-*O*- glucopyranoside (**8**)	12.8 ± 3.7	66.0 [0.8–5.6e4]	—	—
Naringenin-7-*O*- glucopyranoside (**9**)	—	—	28.5 ± 4.6	42.4 [26.6–67.5]
Aromadendrin (**10**)	—	—	67.0 ± 2.6	6 [4.1–8.8]
Myricetin-3-*O*- glucopyranoside (**11**)	30 ± 7.9	184.1 [2.9e-7-1e11]	—	—

MI, maximum inhibition, IC_50_, half maximal inhibitory concentration.

The values in bold represent: Single compounds with a strong inhibitory effect (≥70%).

## 4 Discussion

*E. africana* is a traditional medicinal plant which has been used in different cultures. Different parts of the medicinal plant have been investigated but until now only two flavonoids (apigenin and robonetin) were reported in the roots of the plant (Germanò et al., 2015). Moreover, the leaves of the plant which are used in the Republic of Benin to treat fractures, wounds and sprains have been barely investigated for phenolic compounds. In order to elucidate the phytochemical composition and to assess their bioactivity, the major phenolic compounds were isolated from the extract. The leaves of the plant material were extracted with ethanol 50% (*v/v*) since this resembles the traditional preparation (Marquardt et al., 2020b). The fractionation of the extract by column chromatography on Sephadex^®^ LH-20 was performed to collect groups of compounds which could then be further partitioned. This method is one of the most common methods applied for isolation and purification of various flavonoid derivatives (Mottaghipisheh and Iriti, 2020). The hydroalcoholic leaves extract and fractions thereof were tested for their anti-inflammatory properties *in vitro* using an immortalized keratinocyte cell line in order to assess their influence on proinflammatory cytokine IL-8 and IL-6 release. IL-6 is induced in acute inflammation and triggers the transition from acute to chronic inflammation by enhancing monocyte recruitment (Kaplanski et al., 2003) whereas IL-8 is a chemoattractant mediator of neutrophils which increases expression of surface adhesion molecules and production of ROS at the inflammatory site (Qazi et al., 2011; Ribeiro et al., 2015). The release of both cytokines IL-6 and IL-8 is mediated by two pathways, namely the nuclear factor-κB (NF-κB) and mitogen-activated protein kinase (MAPK) signaling during the proliferation of HaCaT cells (Jae-We et al., 2007; Lee et al., 2019). While normal levels of proinflammatory cytokines prevent infection and accelerate normal wound healing, excessive cytokine production is detrimental as it leads to prolonged inflammation and wound healing. Thus, counteracting proinflammatory cytokine overproduction can mediate a therapeutic effect in chronic wound healing (Xiao et al., 2020). In this study, the crude hydroalcoholic extract exhibited a medium activity on the release of IL-8 with a moderate toxicity towards the HaCaT cell line. The moderate toxicity of the plant extract is presumably due to the presence of rotenone, which has been detected in different parts of the plant and in significant proportions in the roots (Williams, 2012). Although rotenone is a highly lipophilic compound it can appear in a low ratio in an ethanolic extract (Sae-Yun et al., 2006). Further, fractions FC3, FC4, and FC8 from *E. africana* inhibited IL-6 or IL-8 release in a concentration dependent manner. The purification led to 11 compounds out of which 9 were tested. Catechin and gallic were not investigated, since they are frequently occurring molecules with reported anti-inflammatory activity (for reviews see Mena et al., 2014; Bai et al., 2021). Most of the compounds identified in the leaves of *E. africana* are flavonoids which are known to possess different biological properties such as anticancer, antidiabetic, anti-inflammatory, etc. The identified flavonoids are differing in the number of hydroxyl groups, their position and their attachment to a sugar moiety (Wang et al., 2018). 3′,4′,7-trihydroxyflavone, exerted the strongest activity on IL-6 and medium activity on IL-8 release. This trihydroxyflavone belongs to the family of flavonoids and possesses three hydroxyl groups which can interfere with reactive oxygen species and reactive nitrogen species mediated reactions by scavenging free radicals to overcome an oxidative reaction (Gomes et al., 2012). According to various studies, antioxidant and inflammatory effects may be linked through NF-κB activation, which depends sensitively on oxidative stress for the regulation of inflammation (Tuñón et al., 2009). Thus, the demonstrated effect of 3′,4′,7-trihydroxyflavone may be explained by its ability to modulate NF-κB signaling via controlling levels of reactive oxygen species. Besides that, the other structurally similar flavonoid compounds bound to a sugar moiety showed a moderate to mild activity either on the release of IL-6 or IL-8. This binding can reduce the activity of a molecule, which has been demonstrated for most compounds linked to a sugar moiety isolated. The presence of a sugar moiety may decrease the lipophilicity of the molecule and affect its penetration into the cell and its effectiveness. In a previous study, Marquardt et al. (2020a) discussed this observation using the same cell model and testing myricetin-3-*O*-rhamnoside and its aglycone. Besides that, aromadendrin, dihydrokaempferol-7-*O*-glucopyranoside and ethyl gallate exerted a strong to moderate effect on IL-6 release. Thus, an anti-inflammatory effect of *E. africana* and its secondary metabolites may be mediated by a downregulation of proinflammatory cytokine production e.g., in chronic wound healing.

## 5 Conclusion

In the present study, the chemical characterization of phenolic compounds in *E. africana* was reported for the first time and 11 compounds were isolated. 3′,4′,7-trihydroxyflavone exerted strong to moderate inhibitory activity on the release of IL-6 and IL-8 from TNF-α stimulated HaCaT cells. Aromadendrin, dihydrokaempferol-7-*O*-glucoside, and ethyl gallate demonstrated a moderate influence on the release of interleukin IL-6. This finding supports the use of *E. africana* leaves in the traditional medicine for the treatment of inflammatory and pain-related skin conditions in the Republic of Benin.

## Data Availability

The original contributions presented in the study are included in the article/Supplementary Material, further inquiries can be directed to the corresponding author.
