# Supplementary material for: Anti-Inflammatory Potential of Phenolic Compounds Isolated From Entada africana Guill. & Perr. Used in the Republic of Benin
Source: Front Pharmacol. 2022 Jun 30;13:931240. doi: 10.3389/fphar.2022.931240 (PMC9280145; doi:10.3389/fphar.2022.931240)
Supplement: Supplementary file 1 [file DataSheet1.PDF]

Supplementary Material

1 Supplementary Data

Experimental Data of isolated compounds

Gallic acid (**1**): UV (MeCN / H_2O) λ_{max} 218, 271. HRESIMS *m/z* 171.0298 $[M+H]^+$ (calcd. for $C_7H_7O_5$ 171.0288). ^{1}H NMR (CD_3OD, 600 MHz) δ 7.06 (2H, s; H-2 / H-6). ^{13}C NMR (CD_3OD, 151 MHz) δ 170.4 (*C*O), 146.4 (C-3 / C-5), 139.6 (C-4), 122.0 (C-1), 110.3 (C-2 / C-6). Data agreed to those published by (Wang et al., 2015).

Ethylgallate (**2**): UV (MeCN / H_2O) λ_{max} 225, 271. HRESIMS *m/z* 199.0644 $[M+H]^+$ (calcd. for $C_9H_{11}O_5$ 199.0601). ^{1}H NMR (CD_3OD, 600 MHz) δ 7.06 (2H, s, H-2 / H-6), 4.28 (2H, q, *J* = 7.1 Hz, OCH_2), 1.35 (3H, t, *J* = 7.1 Hz, CH_3). ^{13}C NMR (CD_3OD, 151 MHz) δ 168.5 (*C*O), 146.5 (C-3 / C-5), 139.7 (C-4), 121.8 (C-1), 110.0 (C-2 / C-6), 61.7 (OCH_2), 14.6 (CH_3). Data agreed to those published by (Zhao et al., 2009).

5,7-Dihydroxychromone (**3**): UV (MeCN / H_2O) λ_{max} 208, 256, 295. HRESIMS *m/z* 179.0354 $[M+H]^+$ (calcd. for $C_9H_7O_4$ 179.0339). ^{1}H NMR (600 MHz, CD_3OD) δ 7.97 (1H, d, *J* = 5.9 Hz, H-2), 6.34 (1H, d, *J* = 2.1 Hz, H-8), 6.21 (1H, d, *J* = 2.1 Hz, H-6), 6.20 (1H, d, *J* = 5.9 Hz, H-3). ^{13}C NMR (CD_3OD, 151 MHz) δ 183.4 (C-4), 166.2 (C-7), 163.5 (C-5), 159.9 (C-9), 158.1 (C-2), 111.6 (C-3), 106.6 (C-10), 100.2 (C-6), 95.1 (C-8). Data agreed to those published by (Simon et al., 1994).

3′,4′,7-Trihydroxyflavone (**4**): UV (MeCN / H_2O) λ_{max} 211, 236, 341. HRESIMS *m/z* 271.0634 $[M+H]^+$ (calcd. for $C_{15}H_{11}O_5$ 271.0601). ^{1}H NMR (CD_3OD, 600 MHz) δ 7.97 (1H, d, *J* = 8.7 Hz, H-5), 7.41 (1H, dd, *J* = 6.6, 2.2 Hz, H-6′), 7.40 (1H, d, *J* = 2.4 Hz, H-2′), 6.96 (1H, d, *J* = 2.2 Hz, H-8), 6.93 (1H, dd, *J* = 8.4, 2.4 Hz, H-6), 6.91 (1H, d, *J* = 9.2 Hz, H-5′), 6.63 (1H, s, H-3). ^{13}C NMR (CD_3OD, 151 MHz) δ 180.3 (C-4), 166.1 (C-2), 164.9 (C-7), 159.7 (C-9), 150.8 (C-4′), 147.0 (C-3′), 127.8 (C-5), 124.0 (C-1′), 120.2 (C-6′), 117.2 (C-10), 116.8 (C-5′), 116.2 (C-6), 114.1 (C-2′), 105.2 (C-3), 103.5 (C-8). Data agreed to those published by (Júnior et al., 2008; Xu et al., 2018).

Dihydrokaempferol-7-O-β-glucopyranoside (**5**): UV (MeCN / H_2O) λ_{max} 229, 284. HRESIMS *m/z* 451.1246 $[M+H]^+$ (calcd. for $C_{21}H_{23}O_{11}$ 451.1235). ^{1}H NMR (600 MHz, CD_3OD) δ 7.36 (2H, d, *J* = 8.6 Hz, H-2′ / H-6′), 6.83 (2H, d, *J* = 8.6 Hz, H-3′ / H-5′), 6.23 (1H, d, *J* = 2.2 Hz, H-8), 6.21 (1H, d, *J* = 2.2 Hz, H-6), 5.03 (1H, d, *J* = 11.7 Hz, H-2), 4.97 (1H, d, *J* = 7.3 Hz, H-1′′), 4.60 (1H, d, *J* = 11.7 Hz, H-3), 3.87 (1H, dd, *J* = 12.2, 2.2 Hz, H-6a′′), 3.68 (1H, dd, *J* = 12.2, 5.5 Hz, H-6b′′), 3.49 – 3.40 (3H, m, H-2′′ / H-3′′ / H-5′′), 3.40 – 3.36 (1H, m, H-4′′). ^{13}C NMR (151 MHz, CD_3OD) δ 199.4 (C-4), 167.3 (C-7), 164.8 (C-9), 164.3 (C-5) , 159.3 (C-4′), 130.4 (C-2′ / C-6′), 129.1 (C-1′), 116.1 (C-3′ / C-5′), 103.5 (C-10), 101.3 (C-1′′), 98.3 (C-6), 97.0 (C-8), 85.2 (C-2), 78.3 (C-3′′), 77.8 (C-5′′), 74.6 (C-2′′), 73.8 (C-3), 71.1 (C-4′′), 62.3 (C-6′′). Data agreed to those published by (Li and Cui, 2014).

(+)-Catechin (**6**): UV (MeCN / H_2O) λ_{max} 204, 278. HRESIMS *m/z* 291.0875 $[M+H]^+$ (calcd. for $C_{15}H_{15}O_6$ 291.0863). ^{1}H NMR (600 MHz, CD_3OD) δ 6.83 (1H, d, *J* = 1.9 Hz, H-2′), 6.76 (1H, d, *J* = 8.1 Hz, H-5′), 6.72 (1H, dd, *J* = 8.1, 2.0 Hz, H-6′), 5.92 (1H, d, *J* = 2.3 Hz, H-6), 5.85 (1H, d, *J* = 2.3 Hz, H-8), 4.56 (1H. d, *J* = 7.5 Hz, H-2), 3.97 (1H, td, *J* = 7.9, 5.5 Hz, H-3), 2.85 (1H, dd, *J* = 16.1, 5.4

Hz, H-4a), 2.50 (1H, dd, *J* = 16.1, 8.2 Hz, H-4b). Data agreed to those published by (Hori et al., 2018). A carbon spectrum was not recorded due to the low amount of substance.

Quercetin-3-O-[β-apiosyl-(1‴→2″)-β-glucopyranoside] (**7**): UV (MeCN / H_2O) λ_{max} 208, 255, 354. HRESIMS *m/z* 597.1502 [M+H]$^+$ (calcd. for $C_{26}H_{29}O_{16}$ 597.1450). ^{1}H NMR (600 MHz, CD_3OD) δ 7.64 (1H, dd, *J* = 8.3, 2.2 Hz, H-6′), 7.63 (1H, d, *J* = 2.1 Hz, H-2′), 6.87 (1H, d, *J* = 8.3 Hz, H-5′), 6.37 (1H, d, *J* = 2.1 Hz, H-8), 6.18 (1H, d, *J* = 2.1 Hz, H-6), 5.54 (1H, d, *J* = 7.7 Hz, H-1″), 5.47 (1H, d, *J* = 1.3 Hz, H-1‴), 4.04 (1H, d, *J* = 9.6 Hz, H-4a‴), 4.04 (1H, d, *J* = 1.3 Hz, H-2‴), 3.75 (1H, d, *J* = 11.5 Hz, H-5a‴), 3.72 – 3.67 (1H, m, H-6a″), 3.66 (1H, dd, *J* = 9.3, 7.6 Hz, H-2″), 3.63 (1H, d, *J* = 11.5 Hz, H-5b‴), 3.55 (1H, t, *J* = 9.0 Hz, H-3″), 3.51 (1H, dd, *J* = 12.0, 5.6 Hz, H-6b″), 3.32 (1H, overlaid by solvent peak, H-4″), 3.19 (1H, ddd, *J* = 9.9, 5.6, 2.3 Hz, H-5″). ^{13}C NMR (CD_3OD, 151 MHz) δ 179.4 (C-4), 165.8 (C-7), 163.2 (C-5), 158.4 (C-2 / C-9), 149.6 (C-4′), 146.0 (C-3′), 134.9 (C-3), 123.4 (C-1′), 123.3 (C-6′), 117.1 (C-2′), 116.0 (C-5′), 110.6 (C-1‴), 105.9 (C-10), 100.8 (C-1″), 99.7 (C-6), 94.5 (C-8), 80.9 (C-3‴), 78.9 (C-2″), 78.6 (C-3″), 78.3 (C-5″), 78.1 (C-2‴), 75.6 (C-4‴), 71.5 (C-4″), 66.3 (C-5‴), 62.5 (C-6‴). Data agreed to those published by (Kasaj et al., 2001) with a few reassignments at position 2″, 3″ and 5″ according to COSY and HMBC correlations.

Isoquercitrin (Quercetin-3-O-β-glucoside) (**8**): UV (MeCN / H_2O) λ_{max} 218, 255, 354. HRESIMS *m/z* 465.1084 [M+H]$^+$ (calcd. for $C_{21}H_{21}O_{12}$ 465.1028). ^{1}H NMR (CD_3OD, 600 MHz) δ 7.71 (1H, d, *J* = 2.2 Hz, H-2′), 7.59 (1H, dd, *J* = 8.4, 2.2 Hz, H-6′), 6.87 (1H, d, *J* = 8.5 Hz, H-5′), 6.39 (1H, d, *J* = 2.1 Hz, H-8), 6.20 (1H, d, *J* = 2.1 Hz, H-6), 5.25 (1H, d, *J* = 7.7 Hz, H-1″), 3.71 (2H, dd, *J* = 11.9, 2.4 Hz, H-6a″), 3.57 (1H, dd, *J* = 11.9, 5.4 Hz, H-6b″), 3.48 (2H, dd, *J* = 9.2, 7.7 Hz, H-2″), 3.42 (1H, t, *J* = 9.0 Hz, H-3″), 3.35 (1H, t, *J* = 9.6 Hz, H-4″), 3.22 (2H, ddd, *J* = 9.7, 5.4, 2.4 Hz, H-5″). ^{13}C NMR (CD_3OD, 151 MHz) δ 179.4 (C-4), 166.8 (C-7), 163.1 (C-5), 158.9 (C-9), 158.5 (C-2), 149.9 (C-3′), 145.9 (C-4′), 135.6 (C-3), 123.2 (C-1′), 123.1 (C-6′), 117.5 (C-5′), 116.0 (C-2′), 105.5 (C-10), 104.3 (C-1″), 100.1 (C-6), 94.8 (C8), 78.4 (C-5″), 78.1 (C-3″), 75.7 (C-2″), 71.2 (C-4″), 62.6 (C-6″). Data agreed to those published by (Kazuma et al., 2003).

Prunin (Naringenin-7-O-β-glucopyranoside) (**9**): UV (MeCN / H_2O) λ_{max} 230, 281. HRESIMS *m/z* 435.1327 [M+H]$^+$ (calcd. for $C_{21}H_{23}O_{10}$ 435.1286). ^{1}H NMR (CD_3OD, 600 MHz) δ 7.32 (2H, d, *J* = 8.6 Hz, H-2′ / H-6′), 6.81 (2H, d, *J* = 8.6 Hz, H-3′ / H-5′), 6.21 (1H, d, *J* = 2.2 Hz), 6.18 (1H, d, *J* = 2.1 Hz), 5.38 (1H, dt, *J* = 13.0, 3.0 Hz, H-2), 4.96 (1H, d, *J* = 7.1 Hz, H-1″), 3.87 (1H, dd, *J* = 12.2, 2.1 Hz, H-6a″), 3.68 (1H, dd, *J* = 12.2, 5.4 Hz, H-6b″), 3.49 – 3.33 (4H, m, H-2″, H-3″, H-4″, H-5″), 3.17 (1H, dd, *J* = 17.2, 13.0 Hz, H-3a), 2.75 (1H, ddd, *J* = 17.2, 3.1 Hz, H-3b). ^{13}C NMR (151 MHz, CD_3OD) δ 198.6 (C-4), 167.0 (C-7), 165.0 (C-9), 164.6 (C-5), 159.1 (C-4′), 130.9 (C-1′), 129.1 (C-2′ / C-6′), 116.3 (C-3′ / C-5′), 104.9 (C-10), 101.2 (C-1″), 98.0 (C-6), 96.9 (C-8), 80.7 (C-2), 78.3 (C-3″), 77.8 (C-5″), 74.6 (C-2″), 71.1 (C-4″), 62.3 (C-6″), 44.2 (C-3). Data agreed to those published by (Turner et al., 2005).

Aromadendrin (Dihydrokaempferol) (**10**): UV (MeCN / H_2O) λ_{max} 226, 291. HRESIMS *m/z* 289.0739 [M+H]$^+$ (calcd. for $C_{15}H_{13}O_6$ 289.0707). ^{1}H NMR (CD_3OD, 600 MHz) δ 7.35 (2H, d, *J* = 8.4 Hz, H-2′ / H-6′), 6.83 (2H, d, *J* = 8.7 Hz, H-3′ / H-5′), 5.93 (1H, d, *J* = 2.1 Hz, H-8), 5.88 (1H, d, *J* = 2.0 Hz, H-6), 4.98 (1H, d, *J* = 11.6 Hz, H-2), 4.54 (1H, d, *J* = 11.6 Hz, H-3). ^{13}C NMR (CD_3OD, 151 MHz) δ 198.5 (C-4), 168.7 (C-7), 165.3 (C-5), 164.6 (C-9), 159.2 (C-4′), 130.4 (C-2′ / C-6′), 129.3 (C-1′),

116.1 (C-3′ / C-5′), 101.8 (C-10), 97.3 (C-6), 96.3 (C-8), 85.0 (C-2), 73.6 (C-3). Data agreed to those published by (Lee et al., 2003) and (Han et al., 2007).

Myricetin-3-O-glucoside (**11**): UV (MeCN / H_2O) λ_{max} 208, 258, 355. HRESIMS *m/z* 481.0971 $[M+H]^+$ (calcd. for $C_{21}H_{21}O_{13}$ 481.0977). ^{1}H NMR (CD_3OD, 600 MHz) δ 7.30 (2H, s), 6.36 (1H, d, *J* = 2.1 Hz, H-8), 6.18 (1H, d, *J* = 2.1 Hz, H-6), 5.23 (1H, d, *J* = 7.8 Hz, H-1″), 3.73 (1H, dd, *J* = 11.9, 2.4 Hz, H-6a″), 3.62 (1H, dd, *J* = 11.9, 5.2 Hz, H-6b″), 3.51 (1H, dd, *J* = 8.9, 7.9 Hz, H-2″), 3.43 (1H, t, *J* = 8.9 Hz, H-3″), 3.38 (1H, t, *J* = 9.2 Hz, H-4″), 3.23 (1H, ddd, *J* = 9.5, 5.2, 2.4 Hz, H-5″). ^{13}C NMR (CD_3OD, 151 MHz) δ 179.4 (C-4), 166.1 (C-7), 163.1 (C-5), 159.0 (C-2), 158.5 (C-9), 146.4 (C-3′ / C-5′), 135.7 (C-3), 109.9 (C-2′ / C-6′), 105.5 (C-10), 104.6 (C-1″), 100.3 (C-6), 95.2 (C-8), 78.4 (C-5), 78.2 (C-3), 75.7 (C-2), 71.1 (C-4), 62.4 (C-6). Due to the low amount of substance, ^{13}C shifts were additionally established from HSQC and HMBC spectra. Data agreed to those published by (Kazuma et al., 2003).

2 Supplementary Figures and Tables

2.1 Supplementary Figures

Supplementary Figure 1.

TLC Plate1: Fractions 1-8

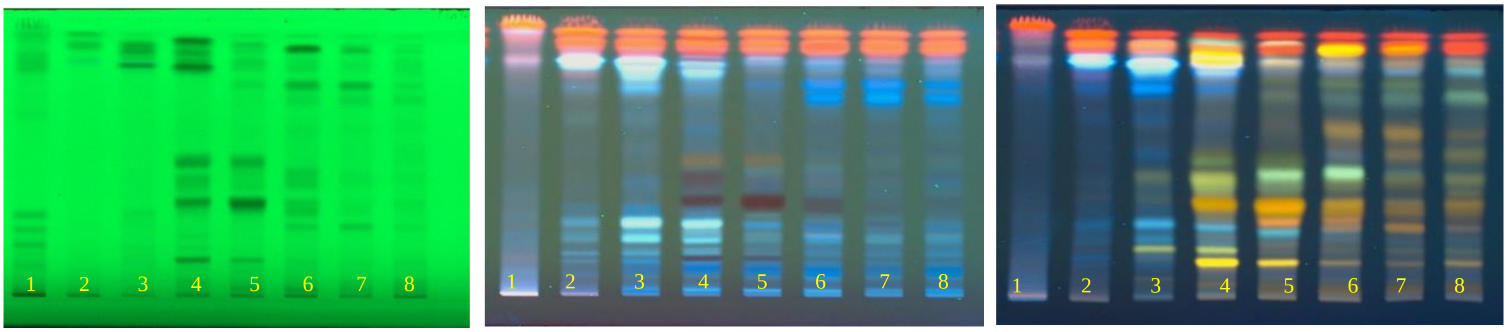

Detection: 254 nm without derivatization

366 nm without derivatization

366 nm after spraying with the Natural product reagent A

TLC Plate 2: Fractions 9-17

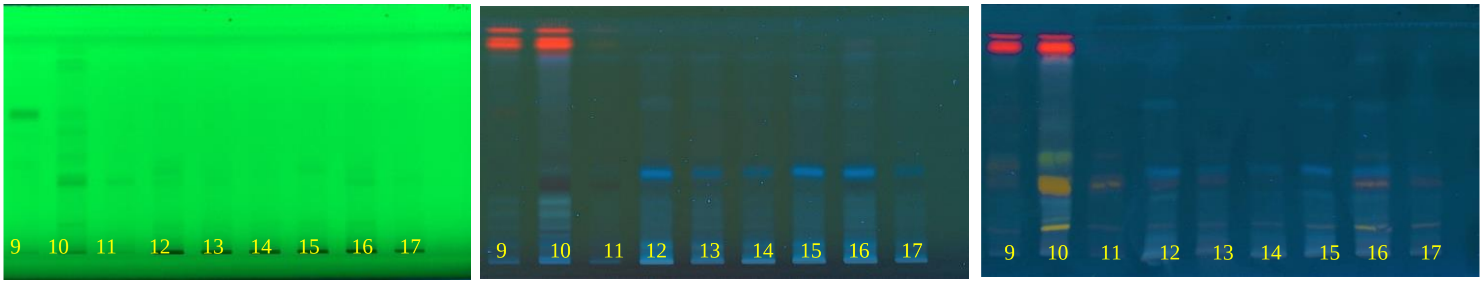

Detection: 254 nm without derivatization

366 nm without derivatization

366 nm after spraying with the Natural product reagent A

TLC Plate 3: Fractions 18-25

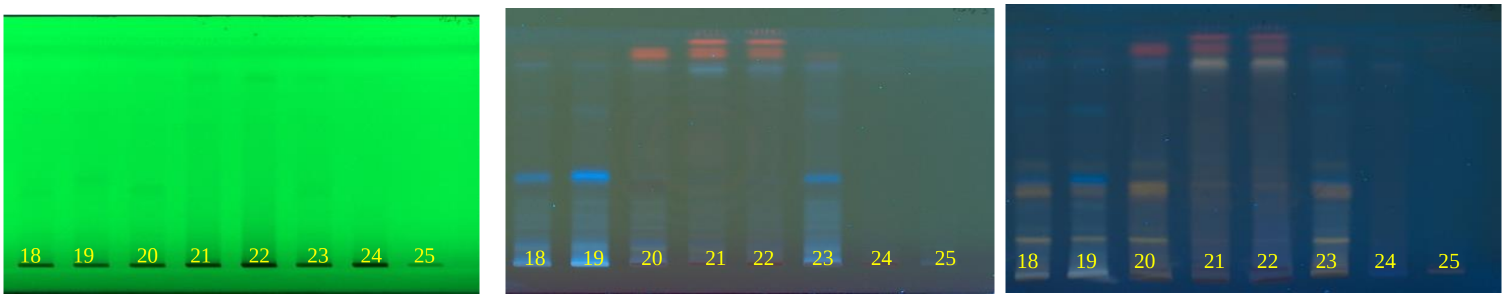

Detection: 254 nm without derivatization

366 nm without derivatization

366 nm after spraying with the Natural product reagent A

Figure S1: TLC profile of the combined fractions obtained by separation of the hydroethanolic leaf extract from *E. africana* on Sephadex® LH-20.

References

Han, X. H., Hong, S. S., Hwang, J. S., Lee, M. K., Hwang, B. Y., and Ro, J. S. (2007). Monoamine oxidase inhibitory components from Cayratia japonica. *Arch. Pharmacal Res.* 30, 13–17. doi: 10.1007/BF02977772.

Hori, K., Wada, M., Yahara, S., Watanabe, T., and Devkota, H. P. (2018). Antioxidant phenolic compounds from the rhizomes of Astilbe rivularis. *Natural Product Research* 32, 453–456. doi:10.1080/14786419.2017.1309536.

Júnior, G. M. V., de M. Sousa, C. M., Cavalheiro, A. J., Lago, J. H. G., and Chaves, M. H. (2008). Phenolic derivatives from fruits of Dipteryx lacunifera Ducke and evaluation of their antiradical activities. *Helv. Chim. Acta* 91, 2159–2167. doi: 10.1002/hlca.200890233.

Kasaj, D., Krenn, L., Gschnell, C., and Kopp, B. (2001). Flavonoid Glycosides from Achillea roseo-albax. *Sci. Pharm.* 69, 211–217. doi: 10.3797/scipharm.aut-01-22.

Kazuma, K., Noda, N., and Suzuki, M. (2003). Malonylated flavonol glycosides from the petals of Clitoria ternatea. *Phytochemistry* 62, 229–237. doi: 10.1016/s0031-9422(02)00486-7

Lee, E. H., Kim, H. J., Song, Y. S., Jin, C., Lee, K.-T., Cho, J., et al. (2003). Constituents of the stems and fruits of Opuntia ficus-indica var. saboten. *Arch. Pharmacal Res.* 26, 1018–1023. doi: 10.1007/BF02994752

Li, C. W., and Cui, C. bin (2014). One new and nine known flavonoids from Choerospondias axillaries and their In Vitro antitumor, anti-hypoxia and antibacterial activities. *Molecules* 19, 21363–21377. doi:10.3390/molecules191221363.

Simon, A., Chulia, A. J., Kaouadji, M., and Delage, C. (1994). Quercetin 3-[triacetylarabinosyl(1→6)galactoside] and chromones from Calluna vulgaris. *Phytochemistry* 36, 1043–1045. doi: 10.1016/S0031-9422(00)90488-6

Turner, A., Chen, S. N., Joike, M. K., Pendland, S. L., Pauli, G. F., and Farnsworth, N. R. (2005). Inhibition of uropathogenic Escherichia coli by cranberry juice: A new antiadherence assay. *Journal of Agricultural and Food Chemistry* 53, 8940–8947. doi:10.1021/jf052035u.

Wang, H. Q., Peng, C. Z., and Chen, Y. G. (2015). Phenolics from Elaeocarpus braceanus. *Chemistry of Natural Compounds* 51, 1167–1168. doi:10.1007/s10600-015-1520-4.

Xu, Y., Tao, Z., Jin, Y., Yuan, Y., Dong, T. T. X., Tsim, K. W. K., et al. (2018). Flavonoids, a Potential New Insight of Leucaena leucocephala Foliage in Ruminant Health. *Journal of Agricultural and Food Chemistry* 66, 7616–7626. doi:10.1021/acs.jafc.8b02739.

Zhao, J., Zhou, X.-W., Chen, X.-B., and Wang, Q.-X. (2009). α-glucosidase inhibitory constituents from Toona sinensis. *Chemistry of Natural Compounds.* 45, 244–246. doi: 10.1007/s10600-009-9289-y
